# Periodic Self-Assembly of Poly(ethyleneimine)–poly(4-styrenesulfonate) Complex Coacervate Membranes

**DOI:** 10.3390/polym15010045

**Published:** 2022-12-22

**Authors:** Ekaterina V. Kukhtenko, Filipp V. Lavrentev, Vladimir V. Shilovskikh, Polina I. Zyrianova, Semyon I. Koltsov, Artemii S. Ivanov, Alexander S. Novikov, Anton A. Muravev, Konstantin G. Nikolaev, Daria V. Andreeva, Ekaterina V. Skorb

**Affiliations:** 1 Infochemistry Scientific Center, ITMO University, 191002 Saint Petersburg, Russia; 2 Department of Materials Science and Engineering, National University of Singapore, Singapore 117575, Singapore

**Keywords:** reaction-diffusion, periodic structure, complex coacervation, polyelectrolytes, Liesegang rings, ion permeability, self-healing

## Abstract

Coacervation is a self-assembly strategy based on the complexation of polyelectrolytes, which is utilized in biomedicine and agriculture, as well as automotive and textile industries. In this paper, we developed a new approach to the on-demand periodic formation of polyelectrolyte complexes through a Liesegang-type hierarchical organization. Adjustment of reaction conditions allows us to assemble materials with a tunable spatiotemporal geometry and establish materials’ production cycles with a regulated periodicity. The proposed methodology allows the membrane to self-assemble when striving to reach balance and self-heal after exposure to external stimuli, such as potential difference and high pH. Using chronopotentiometry, K^+^ ion permeability behavior of the PEI–PSS coacervate membranes was demonstrated. The periodically self-assembled polyelectrolyte nanomembranes could further be integrated into novel energy storage devices and intelligent biocompatible membranes for bionics, soft nanorobotics, biosensing, and biocomputing.

## 1. Introduction

Liesegang rings are periodic bands arising from a controllable precipitation in reaction-diffusion (RD) kinetics. Such modification of interfacial region allows for spatiotemporal self-assembly and disassembly of materials [1,2,3]. Liesegang patterns inspire a novel synthetic technology for programmable spatiotemporally resolved production cycles towards nanostructured materials [4,5,6,7,8], in particular, the nanomaterials for tissue and organ engineering [9]. The reaction partners for Liesegang ring formation now include not only conventional precipitate-forming ions (Ag^+^, Mg^2+^), but also metal–organic frameworks [10,11] and complexes of polymer nanoparticles with organic molecules [12].

Polyelectrolytes are promising candidates for the formation of periodical bands and offer a large number of active groups and dynamic topological structures [13]. The controlled polyelectrolyte organization is particularly interesting for the development of drug delivery agents [14] and batteries [15], as well as smart biocompatible membranes for bionics [16], soft nanorobotics [17], biosensing [18,19], and information storage and biocomputing [20]. However, chemical interactions are several orders of magnitude faster than diffusion in polyelectrolytes, which is a limiting step of polyelectrolyte materials formation [21], and it is particularly important to have charge of chemical kinetics across multiple time scales and achieve precise spatial control over the reaction [22]. Mixing oppositely charged synthetic polyelectrolytes yields spontaneous complexation into hydrated coacervates [23], with solid- or liquid-like properties [24]. Intrinsic properties of coacervates could be suitable for hierarchical organization of multi-PEC systems, where the densest complex is a shell for a liquid coacervate droplet, which opens up avenues to coacervate-only selective adsorption and release of biomolecules [25]. Another approach to heterogeneous systems including coacervates is focused on reversible self-organization of polyelectrolytes into liquid microdroplets. It is based either on spontaneous liquid phase separation [26] or complex hydrophobic-electrostatic surface interactions of coacervate droplets with liposome membranes [27]. Importantly, the formation of PECs is an environmentally friendly water-based approach towards soft functional surfaces [28,29] and nanolayers with tunable microstructure and morphology [30,31,32]; e.g., nanoporous sodium poly(4-styrenesulfonate) (PSS)–poly(allylamine hydrochloride) membranes from water dispersions [33] or PSS–poly(diallyldimethylammonium chloride) coacervate membrane in neutral aqueous media [34].

The morphology, stability, mechanical behavior, and transport properties of polyelectrolyte coacervates can be adjusted by external stimuli (ionic strength, pH, temperature) [35] via impact on the interactions between oppositely charged repeat units in polyelectrolyte complexes (PECs) [36]. Regarding transport properties of polyelectrolyte complexes, recent examples of permeable membranes include nanofiltration membranes for removal of dyes [37], heavy metal ions [38], and micropollutants [39]. High mobility of hydrated chains and dynamic interactions in PECs are also attractive for the development of self-healing materials [40,41].

To the best of our knowledge, there are yet no works on Liesegang ring formation from polyelectrolyte complexes. Here, we propose a facile approach to the formation of functional membranes based on periodic precipitation of PSS and polyethyleneimine (PEI) layers under RD conditions. Through variation of reagent concentrations and molecular weights of polymers, as well as self-assembly methods, the control parameters of membranes’ structure, density, and periodicity were revealed and self-healing behavior after exposure to high pH and voltage, as well as ion permeability were demonstrated.

## 2. Materials and Methods

**Precipitation of PEI–PSS coacervates at a different reactant ratio.** The 2.5 g/L aqueous solutions of PEI (*M*_w_ ≈ 25 kDa) and PSS (*M*_w_ ≈ 70 kDa) were mixed at the weight ratio from 19:1 to 1:19 and doped with 0.1 M NH_4_F.

**Formation of multilayered PEC membranes.** The membranes were prepared in parafilm-sealed Petri dishes from 1 wt% agar gels with 0.1 M NH_4_F and corresponding polyelectrolytes of various concentrations (1.25 g/L, 2.50 g/L, 5.00 g/L) to avoid liquid evaporation. Agar gel was prepared by mixing the agar powder, polyelectrolyte, and NH_4_F in distilled water and subsequent reflux until the mixture became clear. After cooling to room temperature, the solutions were sequentially poured into a selected setup, so that the interlayer between compartments containing oppositely charged polyelectrolytes was filled with an NH_4_F supplemented agar gel.

To perform a one-dimensional model experiment, 4.0 mL of warm agar medium doped with 0.1 M NH_4_F and 2.5 g/L PSS (*M*_w_ ≈ 70 kDa) was transferred to a test tube. The formed gel was covered with 2.0 mL of agar–NH_4_F interlayer and, finally, with 4.0 mL of 0.1 M NH_4_F and 2.5 g/L PEI (*M*_w_ ≈ 25 kDa) dissolved in 1 wt% agar medium.

**Membrane characterization.** The coacervate film growth in agar gel was recorded using a CanoScan 9000F Mark II flatbed scanner (Canon, Tokyo, Japan) in color negative film mode by placing a light-emitting diode behind the sample so that the precipitate appeared in the images as a white shadow. Self-assembly was monitored for over one month, while multilayer films formed from 2.5 g/L polyelectrolyte solutions were recorded at 10 min intervals for 24 h within Petri dishes and for 72 h inside a test tube. Dimensional characteristics of the periodic structures were estimated using ImageJ program.

**Stimuli-responsive disassembly of PEC.** To test the self-healing behavior of the PEC, a piece of hydrogel was cut out from the multilayered PEI–PSS coacervate membrane in a Petri dish followed by addition of 50 μL of 0.1 M NaOH solution to the surface of the mechanically detached fragment. Alternatively, PEI–PSS coacervate membrane in agar-based medium was electrophoretically disassembled by applying the 5 V DC from two graphite electrodes for 1 h, which initiated counterion migration from the PEC phase.

**Ion permeability measurements.** The PEI–PSS coacervate membrane was prepared and tested against permeability of K^+^ ions on a K^+^-selective electrode, fabrication of which is described in detail in [42]. Briefly, the electrochemical cell was based on carbon fiber and consisted of working (WE) and reference electrodes (RE). WE represented a solution of 1.0 wt% valinomycin (potassium ionophore I), 0.5 wt% potassium tetrakis(4-chlorophenyl)borate, 65.5 wt% 2-nitrophenyl octyl ether, and 33.0 wt% high molecular weight poly(vinyl chloride) in 2 mL of 45% THF in H_2_O (all were purchased from Sigma-Aldrich, Burlington, USA) deposited onto polyionic film-modified (eight layers of 2 g/L PEI–PSS) Ag-coated (Kontaktol silver conductive glue, Keller, Saint Petersburg, Russia) carbon fiber with surface density of 250 g m^−2^ (M-Carbo, Minsk, Belarus). The coacervate membrane was formed in a Petri dish after 3 h at the interface between two compartments: the one with PEI + 0.1 M NH_4_F in agar and another one with PSS + 0.1 M NH_4_F in agar. The mass concentrations of polyelectrolytes were 5.00, 2.50, and 1.25 g/L. Then, two round holes were made at equal distances from the membrane. The hole in the compartment with PSS + NH_4_F was filled with deionized water (200 μL, resistivity is 18.6 MΩ), and a two-electrode cell with WE and Ag/AgCl RE, which was pre-conditioned for 12 h in 1M KCl solution, was immersed. A total of 200 μL of 1 M solution of KCl (LenReactiv, Saint Petersburg, Russia) was added to the hole in PEI + NH_4_F compartment. The two-electrode cell was connected to a Potentiostat/Galvanostat SP-50 (Electrochemical Instruments, Chernogolovka, Russia), and chronopotentiograms were recorded under stationary conditions at 23 °C in the current range of 2 A and voltage range of 6 V, with 2 h of conditioning of the cell between permeability measurements. The voltage scan rate was set as 8 points/s.

## 3. Results and Discussion

Spontaneous coacervation of positively and negatively charged polyelectrolytes from dilute solutions is described by Equation (1) [43,44,45,46]:

(1) $${\mathrm{Pol}}^{-}M^{+}\cdot xH_{2}O+{\mathrm{Pol}}^{+}A^{-}\cdot yH_{2}O\to {\mathrm{Pol}}^{+}{\mathrm{Pol}}_{\left(\mathrm{PEC}\right)}^{-}\cdot iH_{2}O+M_{\left(\mathrm{aq}\right)}^{+}+A_{\left(\mathrm{aq}\right)}^{-}+jH_{2}O,$$

where ${\mathrm{Pol}}^{-}$, ${\mathrm{Pol}}^{+}$, $M^{+}$, and $A^{-}$ are polyelectrolyte repeat units and salt ions, respectively, while the (aq) and (PEC) subscripts refer to aqueous solution and coacervate phase [36].

**Figure 1:**
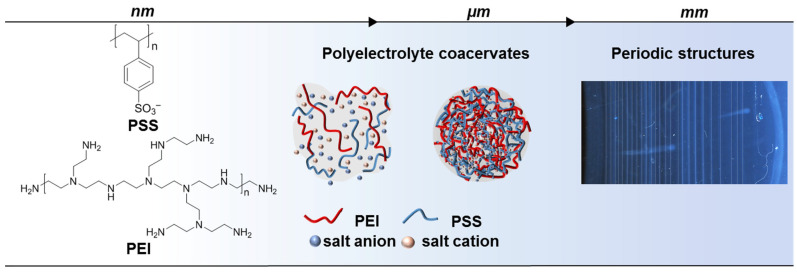
PEC formation at the nano- (nm), micro- (µm) and macro- (mm) scale.

Polyvalent interactions are mainly governed by the entropic release of counterions [47] and H_2_O molecules [48]. Figure 1 shows self-assembly of PEI and PSS macromolecules into coacervate complexes and further into periodic structures. Agar gel was used to control the diffusion rate of polymers, while NH_4_F salt regulated ionic strength of solution and PEI–PSS assembly into micro-coacervates. Visual inspection of polyelectrolytes mixed at various ratios (Figure S1, Table S1) identified that there was a periodic precipitation of stable multilayered polyelectrolyte coacervates at an equimolar polymer ratio.

**Figure 2:**
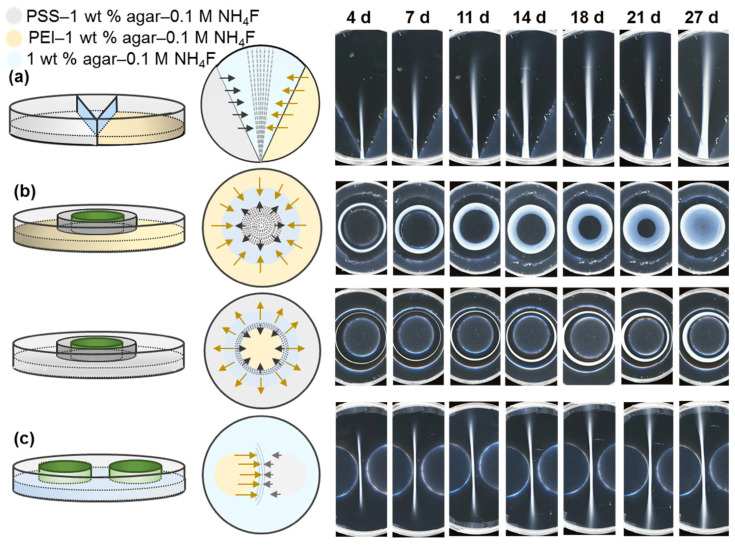
Experimental setups for the formation of diffusion-controlled spatially distributed PEC from PEI (*c* = 2.5 g/L, *M*_w_ ≈ 25 kDa) and PSS (*c* = 2.5 g/L, *M*_w_ *≈* 70 kDa) in 0.1 M NH_4_F: (**a**) tangential periodic bands (method I) (**b**) radial concentric rings (method II) (**c**) semicircles (method III).

Three approaches varying the shape of initial boundary between compartments in Petri dish with the polyelectrolytes were then explored to control their self-organization pattern into membranes of circular and linear geometry. According to method I (Figure 2a), a 90 mm Petri dish was divided into three compartments with two glass slides set at an angle to each other. The side sections were charged with 4.5 mL of agar medium doped with 0.1 M NH_4_F and corresponding polyelectrolyte, while the middle compartment was filled with 8.0 mL of a polyelectrolyte-free gel. In this case, optical photographs show evolution of tangential bands. Method II (Figure 2b) involved formation of three compartments arranged as concentric rings. For this purpose, a 55 mm Petri dish was positioned inside the larger dish so that the outer section could be filled with polyelectrolyte–NH_4_F–agarose medium. After gelation of the solution and removal of the smaller Petri dish, a 33 mm tube flat-top cap was placed in the center of the dish. Subsequently, 5.5 mL of a polyelectrolyte-free gel was transferred into the middle compartment and the cap was withdrawn so that the inner section could be charged with 2.0 mL of an oppositely charged polyelectrolyte agar medium. In this case, a continuous circular membrane is formed due to the radial diffusion of reactants from the peripheral compartment containing PEI to the central one with PSS. The direction of the multilayer growth and the location of precipitate bands suggest a faster mass transfer of PEI as compared to the one of PSS due to the lower molar mass of the PEI. In Method III, the area between two 33 mm tube flat-top caps inside a 90 mm Petri dish was filled with 13.0 mL of polyelectrolyte-free medium (Figure 2c). After gelation of the solution and subsequent removal of the caps, 2.0 mL of a corresponding polyelectrolyte–NH_4_F–agar medium was added to each hole for a gradual formation of semicircular coacervate membrane.

**Figure 3:**
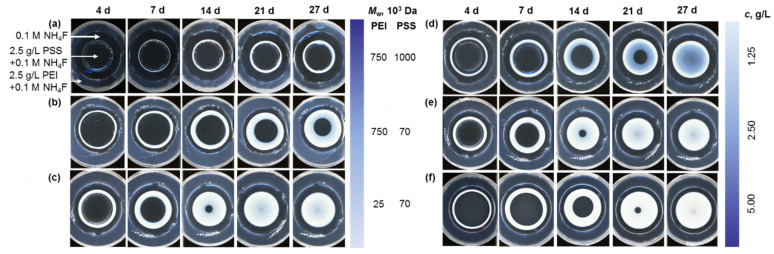
Diffusion-controlled formation of spatially distributed PEC according to *method II* from (**a**–**c**) variable molecular weight 2.5 g/L PEI (*M*_w_ ~ (**a**,**b**) 750 kDa, (**c**) 25 kDa) and 2.5 g/L PSS (*M*_w_ ~ (**a**) 1000 kDa, (**b**,**c**) 70 kDa) in 0.1 M NH_4_F and (**d**–**f**) variable-concentration 25-kDa PEI and 70-kDa PSS (*C* = (**d**) 1.25, (**e**) 2.50, and (**f**) 5.00 g/L).

The membranes were further formed from polyelectrolytes of different average molecular weights: PEI (*M*_w_ ≈ 25 kDa, *M*_w_ ≈ 750 kDa), PSS (*M*_w_ ≈ 70 kDa, *M*_w_ ≈ 1000 kDa) and various concentrations (1.25 g/L, 2.50 g/L, 5.00 g/L) according to *method II*. Figure 3 shows typical morphologies of PEI–PSS membranes obtained by diffusion-controlled precipitation of high and low molecular weight polymers of different concentrations. The topography of the central ring zones of the gel film is represented by an array of circular-symmetric membranes. Coacervate layers broaden from ca. 0.04 to 1.74 mm with an increase in the concentration of polymers, which follows simple empirical laws typical for Liesegang patterns [7]. The layer width also increases with an increase in the molecular weight of the polyelectrolytes due to the abundance of reactive sites and a higher solution viscosity, slowing down the precipitation (Figures S2 and S3). Figure 3 shows that spatiotemporal precipitation of the membranes is highly regular and follows simple empirical laws typical for Liesegang patterns. Time-lapse analysis of the membrane growth according to *method I* and *method II* (Figure S4) shows that the dependence of membrane thickness *r*_n_ on the precipitation time *t*_n_ is described by the time law *r*_n_∝$\sqrt{t_{n}}$ [49] and the mass transfer of the polyelectrolytes within agar gel obeys Fick’s second law (Equation (2)):

(2) $$\frac{dC}{dt}=D\frac{d^{2}C}{dx^{2}},$$

where *D* is a diffusion coefficient, *dx* is the change in the position of the coacervate band from the solution-gel interface, and $\frac{dC}{dt}$ refers to the time gradient of substance concentration. According to Fick’s second law, the rate of subsequent periodic zone formation decreases as the sequence number of membrane increases.

Regardless of the reagent macroscopic properties, there is a linear correlation of the band growth kinetics (Figure S5, Table S2), in perfect agreement with the Jabłczyński spacing law [8,50,51], mainly due to a unidirectional diffusion of the low-molecular-weight component. The spacing law characterizes the increasing distance between consecutive bands and is expressed by the spacing coefficient *p* extracted from the slope of the linear plot of $x_{n+1}$ versus $x_{n}$ (Equation (3)):

(3) $$\frac{x_{n+1}}{x_{n}}\;\approx \;1+p,$$

where $x_{n}$ denotes the location of the *n*th band. Thus, our approach allows a spatiotemporal control over the membrane’s formation.

**Figure 4:**
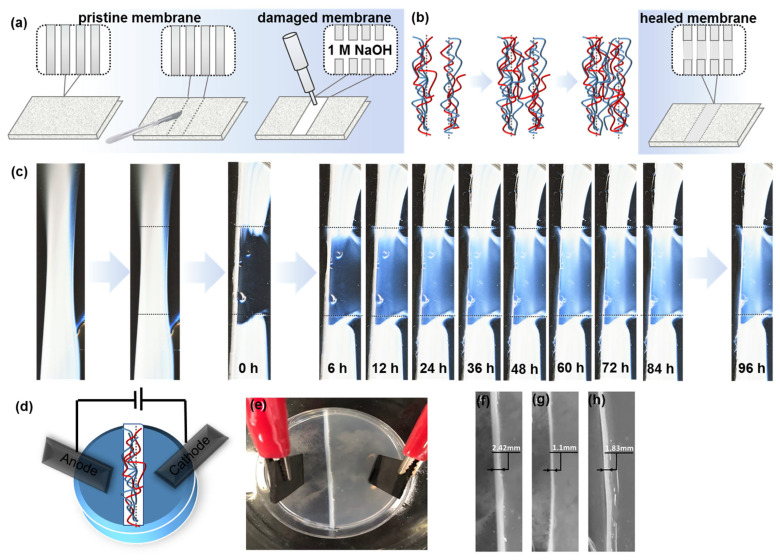
(**a**) Scheme of pristine, damaged, and healed membrane; (**b**) Interdiffusion mechanism of PECs and (**c**) dynamics of its conversion from damaged to self-healed membrane; (**d**) scheme and (**e**) photograph of PEC association-dissociation setup under applied voltage of 5 V and dynamics of self-healing after current cut-off: (**f**) PEC membrane after 24 h of RD self-assembly, *w*_0_ *=* 2.42 mm (**g**) damaged PEC membrane after 1 h of applied voltage, *w*_1_ = 1.1 mm; and (**h**) self-healed PEC membrane, 24 h after maintenance with applied potential gave the thickness of 1.83 mm.

To explore the potential of self-healing membranes, time-lapse analysis of mechanically and chemically damaged periodic materials was carried out. First, two incisions were made at the pristine PEC membrane to cut a hydrogel piece out of the initial macroscopic reaction-diffusion medium. Addition of 50 μL of 0.1 M NaOH to the surface of the mechanically detached fragment (Figure 4a) led to the ready disassembly of periodic coacervate patterns (Figure 4c). After being left in the initial experimental setup, a detached transparent hydrogel sample partially recovered PEC phase with no external intervention. As we see from Figure 4c, hydrogel regeneration began with the rearrangement of mechanically damaged surface and its return to original state, so the torn interfaces were reconnected. After 6 h, PEC re-associated and the material density increased, showing a total recovery (Figure 4c). This was due to the thermodynamically driven interdiffusion of molecules, resulting in the entanglement of polyelectrolyte mobile chains (Figure 4b). However, as oppositely charged polyelectrolytes co-existed in a soluble form within the damaged fragment, no concentration gradients appeared and periodic spatial patterns could not be refabricated.

According to the classical Voorn–Overbeek theory of phase separation in polyelectrolyte solutions [52], electrostatic attractions between oppositely charged species favor coacervate formation. Salt in PECs has been demonstrated to play a role analogous to temperature in polymer melts, enabling long-time relaxation behavior of polyelectrolyte chains [53]. Curtis et al. [54] demonstrated that NaCl (*C* < 150 mM) prevented PEI aggregation and increased solubility. Sufficient salt ions, such as ${\mathrm{NH}}_{4}^{+}$, Na^+^, and F^–^, are available to provide extrinsic charge compensation for charged groups that are not involved in a primarily formed coacervate layer [53,55], preventing its dissolution. This design implies the ability to control the formation and spatial configuration of membrane by applied voltage.

To demonstrate the role of ion pair interactions between polyelectrolyte chains and dissolved salt ions, graphite electrodes were connected to the hydrogel with the PEC membrane (Figure 4d,e). A 5 V potential difference was applied to initial membrane for 1 h, under which ${\mathrm{NH}}_{4}^{+}$ and Na^+^ ions were released from the membrane and moved to cathode, while F^–^ ions moved to anode. After the release of ions, polyelectrolytes that were weakly retained with each other also began to diverge towards electrodes. As a result, the membrane thickness decreases from 2.42 to 1.1 mm (Figure 4f,g). After removal of voltage, polyelectrolyte and ion diffusion partially recovered the membrane thickness up to 1.83 mm within 24 h (Figure 4h). Thus, we suggest that a constant drainage and inflow of ions and polyelectrolytes could provide PEC membranes of any thickness and electrical capacitance values. Different diffusion rates of small ions and polyions to electrode bring the PEC out of equilibrium. The return of the system to equilibrium by the formation of a polyelectrolyte complex takes longer since no external voltage is applied. Thus, we show a self-assembling membrane based on a polyionic hydrogel with programmable properties and electrical stimulus.

**Figure 5:**
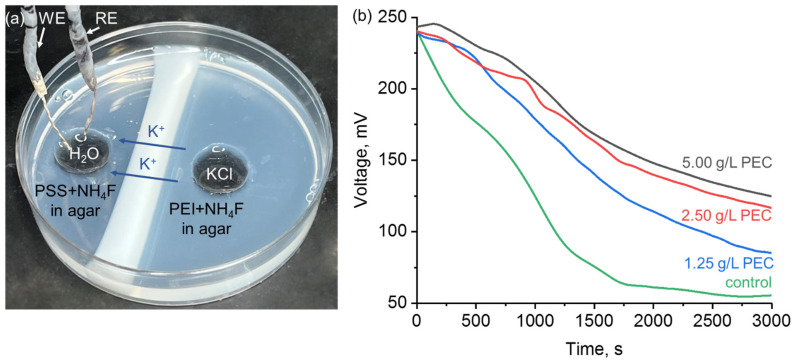
(**a**) Photograph of the electrochemical setup demonstrating K^+^ ion permeability of the PEC membrane (WE = working electrode, RE = reference electrode) and (**b**) chronopotentiograms recorded from K^+^-selective electrode and corresponding to different concentrations of PEC.

The ion permeability of the PEC membranes was finally evaluated against biorelevant K^+^ ions using the setup shown in Figure 5a. The ions of K^+^ are sufficiently large (ionic radius is 1.37 Å) [56] and could reveal the limiting permeability of polyelectrolyte membrane towards metal ions. Chronopotentiograms recorded without PEC membrane in agar+NH_4_F medium (control, Figure 5b) show a gradual decrease in voltage from ca. 250 mV to 50 mV within ca. 3 h. Once the membrane separates PEI- and PSS-containing compartments, the final voltage value increases to ca. 100 mV (at 1.25 g/L PEC) to 130 mV (2.50 and 5.00 g/L PEC). This result indicates that the concentration of 1.25 g/L of the PEC in the membrane is sufficient for K^+^ ion permeability. Whereas an increase in the concentration of polyelectrolytes seems to decrease the membrane’s permeability presumably due to the restricted ion transfer through the polyelectrolyte-based hydrogel arising from ion binding via amino groups in PEI–PSS coacervate [57].

The above results demonstrate that the PEI–PSS coacervate complex represents the membrane with the self-healing ability and ion permeability towards K^+^ ions. It should be noted that the ion permeability did not significantly alter in the case of the nanomembranes recovered to initial state after exposure to high pH or potential difference. Therefore, the suggested nanomembranes could further be integrated into novel energy storage devices and intelligent biocompatible membranes for bionics, soft nanorobotics, biosensing, and biocomputing.

## 4. Conclusions

A fast and facile approach to the periodic formation of polyelectrolyte membranes has been developed. The novel synthetic technology for membranes’ production mimics the diffusion-controlled precipitation of Liesegang rings. The adjustment of reaction conditions, such as the composite concentrations, ratio and polymer molecular weight, allows us to program periodicity and production cycles on demand. The stimuli-responsive membrane is capable of self-assembly and self-disassembly when a potential difference is applied. An electrostimulated polyionic membrane based on a hydrogel makes it possible to create a new generation of biomimetic devices. pH-sensitivity and self-healing behavior of our periodic materials propel the development of structure- and composition-controllable stimuli responsive membranes that are integrable into soft-nanorobotic devices, energy storage devices, actuators, sensors and drug delivery systems to meet the demands of biomaterials engineering.

## Data Availability

Original data of this work are given in Supplementary Materials.

## Supplementary Materials

The following supporting information can be downloaded at: https://www.mdpi.com/article/10.3390/polym15010045/s1, Figure S1: Evolution of PEI–PSS complex coacervate, *M*_w_ (PEI) ≈ 25 kDa, *M*_w_ (PSS) ≈ 70 kDa, at various PEI:PSS ratio (a) immediately after the precipitation and (b) in 4 days. 1) 9:1; (2) 17:3; (3) 4:1; (4) 3:1; (5) 7:3; (6) 13:7; (7) 3:2; (8) 11:9; (9) 1:1; (10) 9:11; (11) 2:3; Figure S2: Diffusion-controlled spatial distribution of a complex coacervate obtained from PEI (*M*_w_ ≈ 750 kDa) and PSS (*M*_w_ *≈* 1000 kDa) in 0.1 M NH_4_F using methods (a,c–e) I and (b,f) III. *C*_PEI_ = *C*_PSS_ = (a,b) 1.25 g/L, (c) 2.50 g/L, and (d) 5.00 g/L; (e,f) *C*_PEI_ = 2.50 g/L, *C*_PSS_ = 1.25 g/L; Figure S3: Diffusion-controlled spatial distribution of a complex coacervate obtained according to method (b,c,i) I, (a,d,e) II, and (f,h,k) III from PEI and PSS (*M*_w_ ≈ 70 kDa) in 0.1 M NH_4_F. *M*_w_ (PEI) ≈ (a,b) 750 kDa, (c–k) 25 kDa; *C*_PEI_ = *C*_PSS_ = (a,b,e,f) 2.50 g/L; (c,d) 1.25 g/L, and (g,h) 5.00 g/L; (i) *C*_PEI_ = 2.50 g/L, *C*_PSS_ = 1.25 g/L; (k) *C*_PEI_ = 2.50 g/L, *C*_PSS_ = 1.25 g/L; Figure S4: Kymograph-like space–time plots of a diffusion-controlled PEI–PSS complex coacervate formation, *M*_w_ (PEI) ≈ 25 kDa, *M*_w_ (PSS) ≈ 70 kDa, *C*_PEI_ = *C*_PSS_ = 2.5 g/L in 0.1 M NH_4_F: (a) semicircles (*method III*) setup (b) tangential periodic bands (*method I*) setup; Figure S5: Diffusion-controlled spatial distribution of PEI–PSS complex coacervate in 0.1 M NH_4_F and the ratio of the neighboring precipitate bands position. (a–d) *M*_w_ (PEI) ~ 750 kDa, *M*_w_ (PSS) ~ 1000 kDa; (e–h) *M*_w_ (PEI) ≈ 25 kDa, *M*_w_ (PSS) ≈ 70 kDa. *C*_PEI_ = *C*_PSS_ = (a,c,e) 1.25 g/L; (b,f) 2.50 g/L, and (d,h) 5.00 g/L; (c,g) *C*_PEI_ = 2.50 g/L, *C*_PSS_ = 1.25 g/L; Table S1: Appearance of 25-kDa PEI–70-kDa PSS coacervate at various PEI:PSS ratio; Table S2: Statistical analysis of diffusion-controlled spatial distribution of PEI–PSS complex coacervate from Figure S5.
