# Supplementary material for: Periodic Self-Assembly of Poly(ethyleneimine)–poly(4-styrenesulfonate) Complex Coacervate Membranes
Source: Polymers (Basel). 2022 Dec 22;15(1):45. doi: 10.3390/polym15010045 (PMC9824353; doi:10.3390/polym15010045)
Supplement: Supplementary file 1 [file polymers-15-00045-s001.zip › polymers-2002153-supplementary.pdf]

Supporting Information for:

Periodic Self-Assembly of poly(ethyleneimine)–poly(4-styrenesulfonate) Complex Coacervate Membranes

Ekaterina V. Kukhtenko [1], Filipp V. Lavrentev [1], Vladimir V. Shilovskikh [1], Polina I. Zyrianova [1], Semyon I. Koltsov [1], Artemii S. Ivanov [1], Alexander S. Novikov [1], Anton A. Muravev [1], Konstantin G. Nikolaev [1], Daria V. Andreeva [2] and Ekaterina V. Skorb [1,*]

[1] Infochemistry Scientific Center, ITMO University, 191002 Saint Petersburg, Russia
[2] Department of Materials Science and Engineering, National University of Singapore, Singapore 117575, Singapore
[*] Correspondence: skorb@itmo.ru

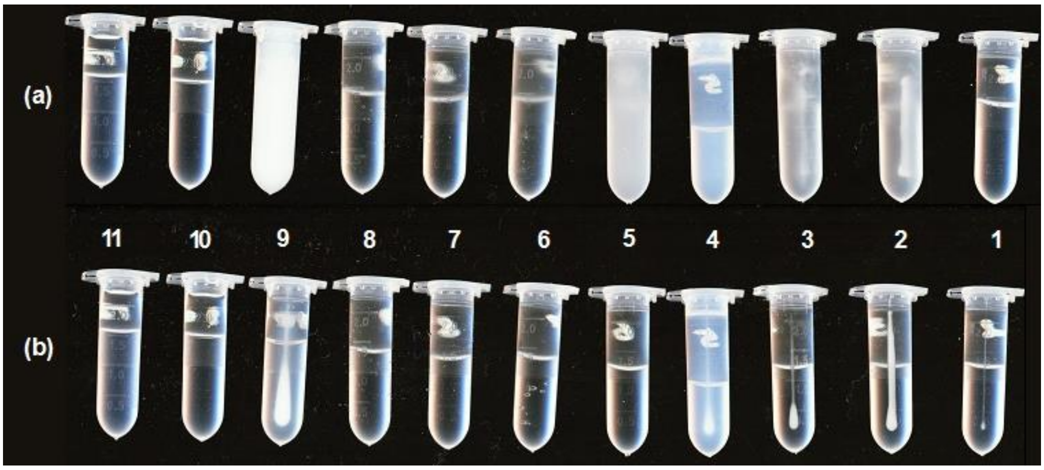

Figure S1. Evolution of PEI–PSS complex coacervate, M_w (PEI) ≈ 25 kDa, M_w (PSS) ≈ 70 kDa, at various PEI : PSS ratio (a) immediately after the precipitation and (b) in 4 days. 1) 9:1; (2) 17:3; (3) 4:1; (4) 3:1; (5) 7:3; (6) 13:7; (7) 3:2; (8) 11:9; (9) 1:1; (10) 9:11; (11) 2:3.

Table S1. Appearance of 25-kDa PEI–70-kDa PSS coacervate at various PEI : PSS ratio

C_{PEI}, g/L	C_{PSS}, g/L	PEI : PSS ratio	Result
2.38	0.13	19 : 1	No coacervation
2.25	0.25	9 : 1	Traces of coacervate, slow precipitation.
2.13	0.38	17 : 3	
2.00	0.50	4 : 1	Readily formed soft opalescent precipitate (stable in 4 d)
1.88	0.63	3 : 1	
1.75	0.75	7 : 3	Readily formed dense precipitate (dissolves within 4 d)
1.63	0.88	13 : 7	
1.50	1.00	3 : 2	No coacervation
1.38	1.13	11 : 9	
1.25	1.25	1 : 1	Ready abundant formation of a dense precipitate (stable in 4 d)
1.13	1.38	9 : 11	
1.00	1.50	2 : 3	
0.88	1.63	7 : 13	
0.75	1.75	3 : 7	
0.63	1.88	1 : 3	No coacervation
0.50	2.00	1 : 4	
0.38	2.13	3 : 17	
0.25	2.25	1 : 9	
0.13	2.38	1 : 19	

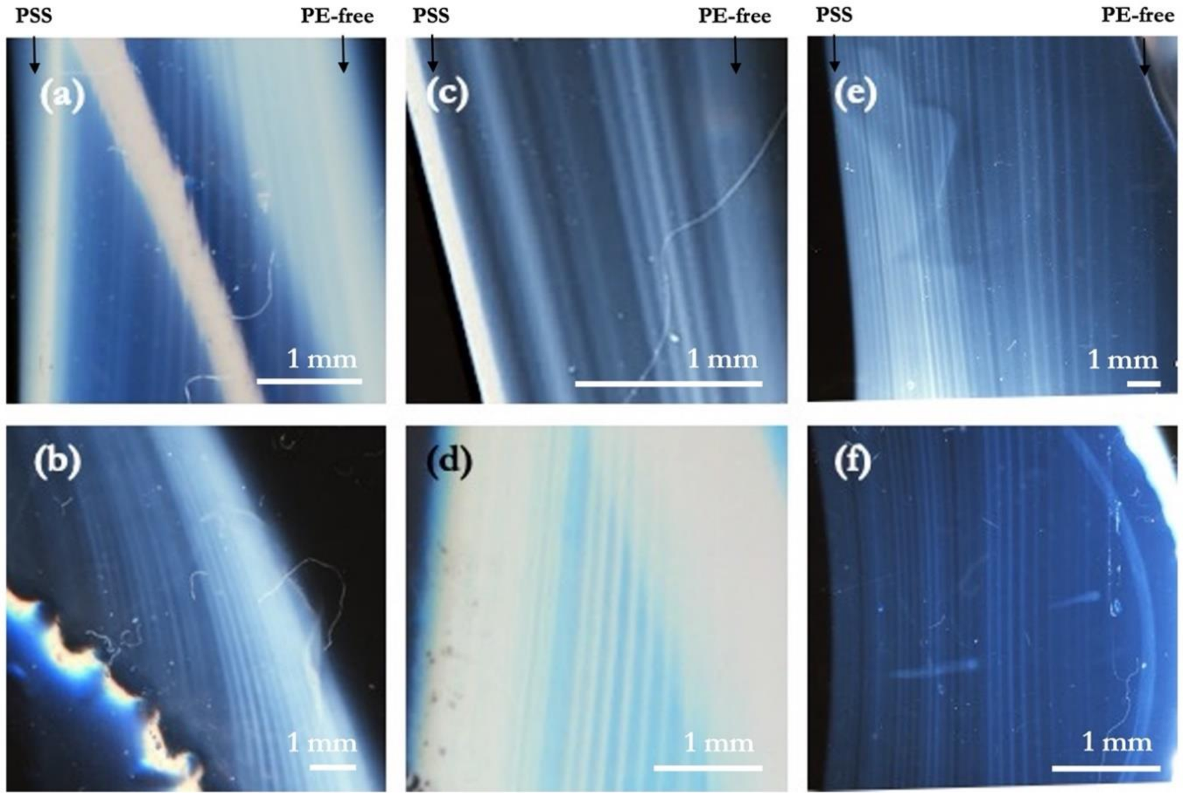

Figure S2. Diffusion-controlled spatial distribution of a complex coacervate obtained from PEI (M_w ≈ 750 kDa) and PSS (M_w ≈ 1000 kDa) in 0.1 M NH_4F using methods (a,c–e) I and (b,f) III. C_{PEI} = C_{PSS} = (a,b) 1.25 g/L, (c) 2.50 g/L, and (d) 5.00 g/L; (e,f) C_{PEI} = 2.50 g/L, C_{PSS} = 1.25 g/L.

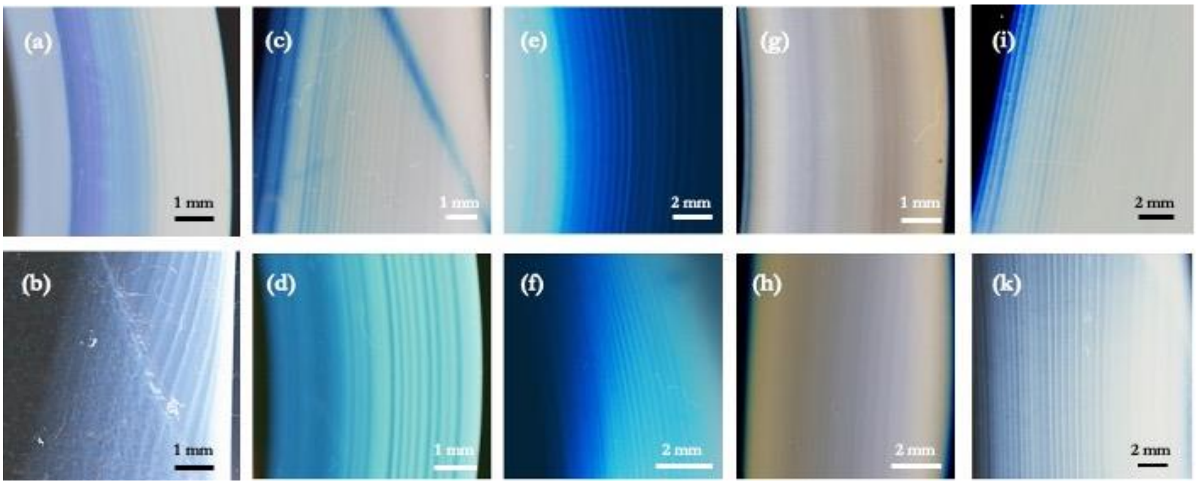

Figure S3. Diffusion-controlled spatial distribution of a complex coacervate obtained according to method (b,c,i) I, (a,d,e) II, and (f,h,k) III from PEI and PSS (M_w ≈ 70 kDa) in 0.1 M NH_4F. M_w (PEI) ≈ (a,b) 750 kDa, (c–k) 25 kDa; C_{PEI} = C_{PSS} = (a,b,e,f) 2.50 g/L; (c,d) 1.25 g/L, and (g,h) 5.00 g/L; (i) C_{PEI} = 2.50 g/L, C_{PSS} = 1.25 g/L; (k) C_{PEI} = 2.50 g/L, C_{PSS} = 1.25 g/L.

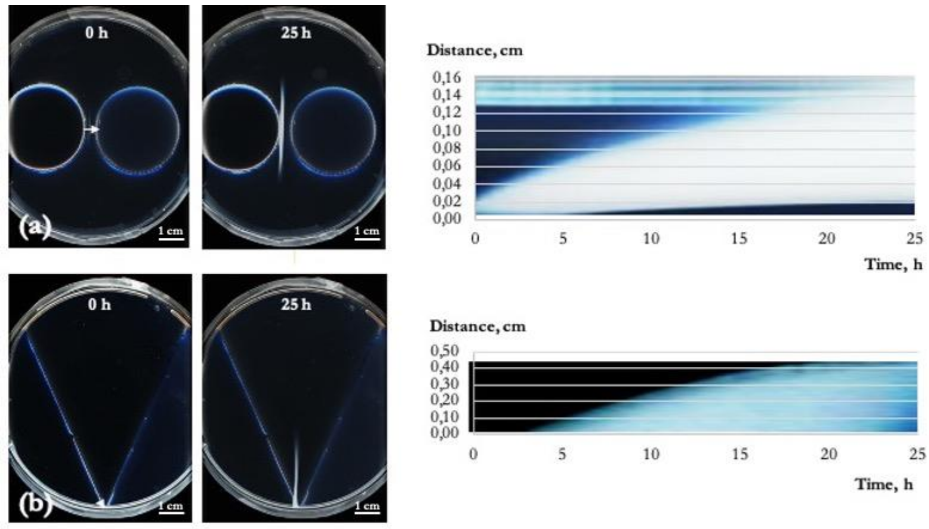

Figure S4. Kymograph-like space–time plots of a diffusion-controlled PEI–PSS complex coacervate formation, M_w (PEI) ≈ 25 kDa, M_w (PSS) ≈ 70 kDa, $C_{PEI} = C_{PSS}$ = 2.5 g/L in 0.1 M NH_4F: (a) semicircles (*method III*) setup (b) tangential periodic bands (*method I*) setup.

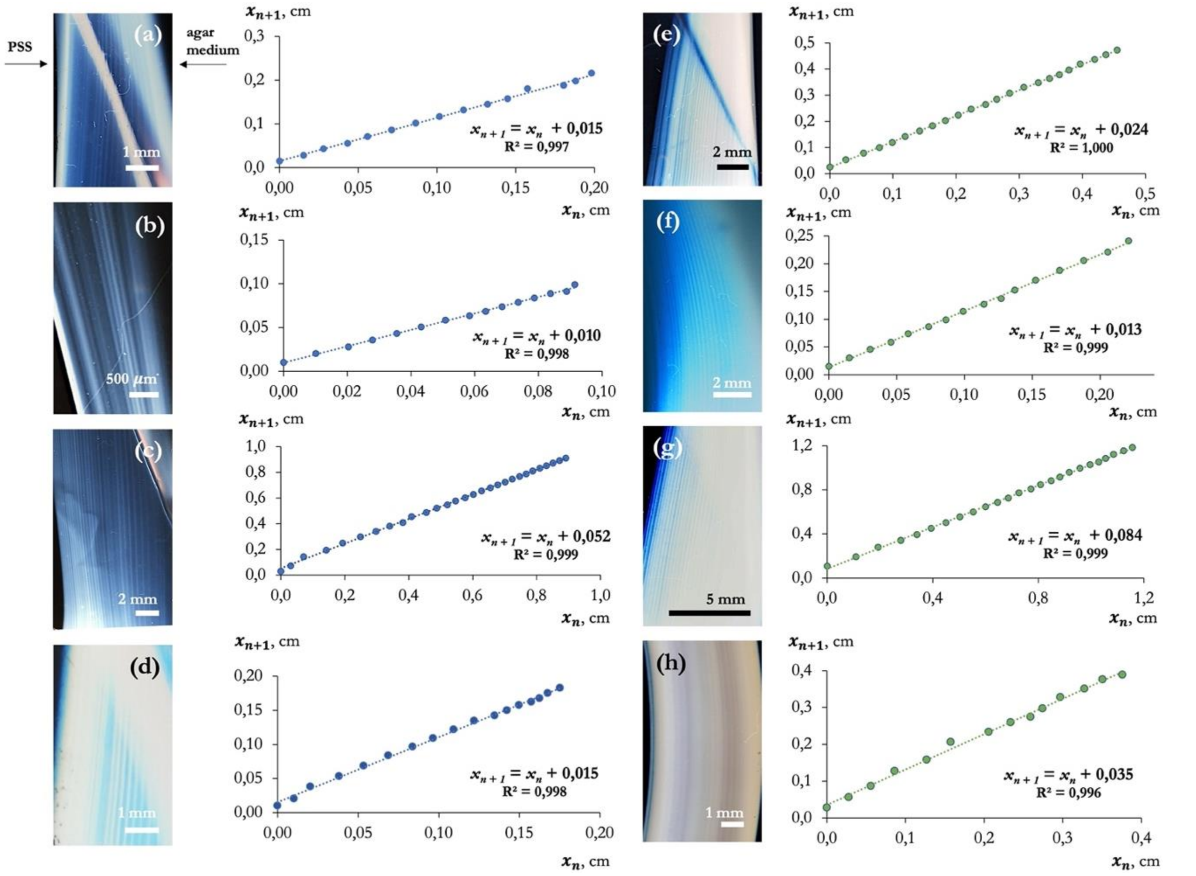

Figure S5. Diffusion-controlled spatial distribution of PEI–PSS complex coacervate in 0.1 M NH_4F and the ratio of the neighboring precipitate bands position. (a–d) M_w (PEI) ≈ 750 kDa, M_w (PSS) ≈ 1000 kDa; (e–h) M_w (PEI) ≈ 25 kDa, M_w (PSS) ≈ 70 kDa. $C_{PEI} = C_{PSS}$ = (a,c,e) 1.25 g/L; (b,f) 2.50 g/L, and (d,h) 5.00 g/L; (c,g) C_{PEI} = 2.50 g/L, C_{PSS} = 1.25 g/L.

Table S2. Statistical analysis of diffusion-controlled spatial distribution of PEI–PSS complex coacervate from Figure S5

Entry from Figure S5a	**x_n, cm**	**x_{n+1}, cm**	*Δ, cm*	*P = Δ (n+1) / Δ n*
1	0.000	0.015	0.015	1.200
2	0.015	0.028	0.013	0.833
3	0.028	0.043	0.015	1.200
4	0.043	0.056	0.013	0.833
5	0.056	0.071	0.015	1.000
6	0.071	0.086	0.015	1.000
7	0.086	0.102	0.015	1.000
8	0.102	0.117	0.015	1.000
9	0.117	0.132	0.015	1.200
10	0.132	0.145	0.013	1.000
11	0.145	0.157	0.013	0.556
12	0.157	0.180	0.023	3.000
13	0.180	0.188	0.008	0.750
14	0.188	0.198	0.010	0.571
15	0.198	0.216	0.018	2.333
16	0.216	0.224	0.008	
			**Mean**	1.165
			**S.D.**	0.63

Entry from Figure S5b	**x_n, cm**	**x_{n+1}, cm**	*Δ, cm*	*P = Δ (n+1) / Δ n*
1	0.000	0.010	0.010	1.000
2	0.010	0.020	0.010	1.333
3	0.020	0.028	0.008	1.000
4	0.028	0.036	0.008	1.000
5	0.036	0.043	0.008	1.000
6	0.043	0.051	0.008	1.000
7	0.051	0.058	0.008	1.500
8	0.058	0.064	0.005	1.000
9	0.064	0.069	0.005	1.000
10	0.069	0.074	0.005	1.000
11	0.074	0.079	0.005	1.000
12	0.079	0.084	0.005	1.000
13	0.084	0.089	0.005	2.000
14	0.089	0.091	0.003	0.333
15	0.091	0.099	0.008	
			**Mean**	1.083
			**S.D.**	0.35

Entry from Figure S5c	***x_n, cm***	***x_{n+1}, cm***	*Δ, cm*	*P = Δ (n+1) / Δ n*
1	0.000	0.030	0.030	0.750
2	0.030	0.071	0.041	0.571
3	0.071	0.142	0.071	1.400
4	0.142	0.193	0.051	0.909
5	0.193	0.249	0.056	1.158
6	0.249	0.297	0.048	1.118
7	0.297	0.340	0.043	1.063
8	0.340	0.381	0.041	1.455
9	0.381	0.409	0.028	0.611
10	0.409	0.455	0.046	1.385
11	0.455	0.488	0.033	1.000
12	0.488	0.521	0.033	1.300
13	0.521	0.546	0.025	0.833
14	0.546	0.577	0.030	1.200
15	0.577	0.602	0.025	1.000
16	0.602	0.627	0.025	0.909
17	0.627	0.655	0.028	1.222
18	0.655	0.678	0.023	1.000
19	0.678	0.701	0.023	1.000
20	0.701	0.724	0.023	1.000
21	0.724	0.747	0.023	1.125
22	0.747	0.767	0.020	1.000
23	0.767	0.787	0.020	0.889
24	0.787	0.810	0.023	1.125
25	0.810	0.831	0.020	1.000
26	0.831	0.851	0.020	1.000
27	0.851	0.871	0.020	1.000
28	0.871	0.892	0.020	1.143
29	0.892	0.909	0.018	
			**Mean**	1.042
			**S.D.**	0.21

Entry from Figure S5d	***x_n, cm***	***x_{n+1}, cm***	*Δ, cm*	*Δ (n+1) / Δ n*
1	0.000	0.010	0.010	1.000
2	0.010	0.020	0.010	0.571
3	0.020	0.038	0.018	1.167
4	0.038	0.053	0.015	1.000
5	0.053	0.069	0.015	1.000
6	0.069	0.084	0.015	1.200
7	0.084	0.097	0.013	1.000
8	0.097	0.109	0.013	1.000
9	0.109	0.122	0.013	1.000

10	0.122	0.135	0.013	1.667
11	0.135	0.142	0.008	1.000
12	0.142	0.150	0.008	1.000
13	0.150	0.157	0.008	1.500
14	0.157	0.163	0.005	1.000
15	0.163	0.168	0.005	0.667
16	0.168	0.175	0.008	1.000
17	0.175	0.183	0.008	
			**Mean**	1.048
			**S.D.**	0.25

Entry from Figure S5e	***x_n, cm***	***x_{n+1}, cm***	*Δ, cm*	*P = Δ (n+1) / Δ n*
1	0.000	0.025	0.025	0.909
2	0.025	0.053	0.028	1.100
3	0.053	0.079	0.025	1.250
4	0.079	0.099	0.020	1.000
5	0.099	0.119	0.020	0.889
6	0.119	0.142	0.023	1.125
7	0.142	0.163	0.020	1.000
8	0.163	0.183	0.020	1.000
9	0.183	0.203	0.020	1.000
10	0.203	0.224	0.020	0.889
11	0.224	0.246	0.023	1.286
12	0.246	0.264	0.018	0.875
13	0.264	0.284	0.020	0.889
14	0.284	0.307	0.023	1.000
15	0.307	0.330	0.023	1.286
16	0.330	0.348	0.018	1.167
17	0.348	0.363	0.015	1.000
18	0.363	0.378	0.015	0.857
19	0.378	0.396	0.018	0.778
20	0.396	0.419	0.023	1.286
21	0.419	0.437	0.018	1.000
22	0.437	0.455	0.018	1.000
23	0.455	0.472	0.018	0.700
			**Mean**	1.012
			**S.D.**	0.16

Entry from Figure S5f	***x_n, cm***	***x_{n+1}, cm***	*Δ, cm*	*P = Δ (n+1) / Δ n*
1	0.000	0.015	0.015	1.000
2	0.015	0.030	0.015	1.000
3	0.030	0.046	0.015	1.200
4	0.046	0.058	0.013	0.833

5	0.058	0.074	0.015	1.200
6	0.074	0.086	0.013	1.000
7	0.086	0.099	0.013	0.833
8	0.099	0.114	0.015	1.200
9	0.114	0.127	0.013	1.250
10	0.127	0.137	0.010	0.667
11	0.137	0.152	0.015	0.857
12	0.152	0.170	0.018	1.000
13	0.170	0.188	0.018	1.000
14	0.188	0.206	0.018	1.167
15	0.206	0.221	0.015	0.750
16	0.221	0.241	0.020	
			**Mean**	0.997
			**S.D.**	0.18

Entry from Figure S5g	x_n, **cm**	x_{n+1}, **cm**	*Δ, cm*	*P = Δ (n+1) / Δ n*
1	0.000	0.109	0.109	1.303
2	0.109	0.193	0.084	0.971
3	0.193	0.279	0.086	1.417
4	0.279	0.340	0.061	1.143
5	0.340	0.394	0.053	0.955
6	0.394	0.450	0.056	1.048
7	0.450	0.503	0.053	1.050
8	0.503	0.554	0.051	1.111
9	0.554	0.599	0.046	1.000
10	0.599	0.645	0.046	1.125
11	0.645	0.686	0.041	1.000
12	0.686	0.726	0.041	0.889
13	0.726	0.772	0.046	1.286
14	0.772	0.808	0.036	0.875
15	0.808	0.848	0.041	1.231
16	0.848	0.881	0.033	0.929
17	0.881	0.917	0.036	0.875
18	0.917	0.958	0.041	1.067
19	0.958	0.996	0.038	1.154
20	0.996	1.029	0.033	1.300
21	1.029	1.054	0.025	0.833
22	1.054	1.085	0.030	0.800
23	1.085	1.123	0.038	1.154
24	1.123	1.156	0.033	1.182
25	1.156	1.184	0.028	
			**Mean**	1.071
			**S.D.**	0.16

Entry from Figure S5h	***x_n, cm***	***x_{n+1}, cm***	*Δ, cm*	*P = Δ (n+1) / Δ n*
1	0.000	0.028	0.028	1.000
2	0.028	0.056	0.028	0.917
3	0.056	0.086	0.030	0.750
4	0.086	0.127	0.041	1.333
5	0.127	0.157	0.030	0.632
6	0.157	0.206	0.048	1.727
7	0.206	0.234	0.028	1.100
8	0.234	0.259	0.025	1.667
9	0.259	0.274	0.015	0.667
10	0.274	0.297	0.023	0.750
11	0.297	0.328	0.030	1.333
12	0.328	0.351	0.023	0.900
13	0.351	0.376	0.025	2.000
14	0.376	0.389	0.013	
			**Mean**	1.137
			**S.D.**	0.43
